# Acute Respiratory Disease in US Army Trainees 3 Years after Reintroduction of Adenovirus Vaccine^1^

**DOI:** 10.3201/eid2301.161297

**Published:** 2017-01

**Authors:** Nakia S. Clemmons, Zachary D. McCormic, Joel C. Gaydos, Anthony W. Hawksworth, Nikki N. Jordan

**Affiliations:** US Army Public Health Center, Aberdeen Proving Ground, Maryland, USA (N.S. Clemmons, Z.D. McCormic, J.C. Gaydos, N.N. Jordan);; US Naval Health Research Center, San Diego, California, USA (A.W. Hawksworth)

**Keywords:** adenovirus, US Army trainees, acute respiratory disease, ARD, vaccine, vaccination, disease rates, viruses, outbreaks, high-risk population, dynamic population, basic training, initial entry training, military

## Abstract

The 1999 cessation of vaccination against adenovirus types 4 and 7 among US Army trainees resulted in reemergence of acute respiratory disease (ARD) outbreaks. The 2011 implementation of a replacement vaccine led to dramatic and sustained decreases in ARD cases, supporting continuation of vaccination in this population at high risk for ARD.

In the past, febrile acute respiratory disease (ARD) was a major cause of illness among military members, especially those in initial entry training (IET [basic training]), a physically and mentally demanding 6- to 12-week program (*1*–*5*). Most cases were caused by infection with adenovirus types 4 and 7; 80% of trainees became infected and 20% were hospitalized (*5*).

Routine use of oral adenovirus type 4 and 7 (AdV-4 and -7) vaccine began in 1971 and eventually included year-round vaccination, resulting in plummeting ARD rates (*1*). In 1994, the sole vaccine manufacturer stopped production. The last doses were shipped in 1996 and administered only during winter until stocks were depleted in 1999; ARD rates subsequently increased at IET sites (*3*,*4*).

When the stock of AdV-4 and -7 vaccine was depleted, the Army’s Acute Respiratory Disease Surveillance Program (ARD-SP), partnering with the Naval Health Research Center (San Diego, CA, USA) Febrile Respiratory Illness (NHRC FRI) Surveillance Program, demonstrated substantial increases in ARD cases, specifically adenovirus-associated ARD. These cases cost ≈$10–$26 million each year in medical care and lost recruit time (*5*). In addition, a threat existed for the emergence of other adenovirus types that could cause severe and fatal disease (*5*). In March 2011, a new, 2-tablet, live, enteric-coated oral AdV-4 and -7 vaccine was licensed by the US Food and Drug Administration for use in US military members. Administration of the vaccine to trainees early in their IET program began in November 2011 and reached full coverage of all trainees by year’s end. In 2014, NHRC reported that, after 2 years of AdV-4 and -7 vaccine use, a dramatic decline was seen in febrile ARD cases in training centers across the military services, and no indication was seen of a serious, sustained emergence of a new adenovirus threat (*5*). We report the ARD-SP and NHRC FRI data for the US Army IET population during the first 3 years after reintroduction of AdV-4 and -7 vaccine, looking at variations in ARD rates at 4 Army IET sites and at adenovirus types identified in trainees with ARD.

## The Study

In 1966, the ARD-SP, then called the Adenovirus Surveillance Program, was implemented to monitor ARD and evaluate the new AdV-4 and -7 vaccine at IET sites (*2*). In 1996, partly in response to increasing ARD cases, the NHRC initiated the FRI Surveillance Program to assess febrile respiratory illness rates, etiologies, and trends across military training installations (*5*). ARD-SP captured all ARD cases, and NHRC FRI collected respiratory specimens from a convenience sample of the ARD-SP cohort. This subset was tested for respiratory pathogens, including adenoviruses. Together, the ARD-SP, operated by the Army Public Health Center (Aberdeen Proving Ground, MD), and the NHRC FRI program have provided coordinated surveillance of respiratory pathogens for Army IET populations.

We studied aggregate data from 2010–2014 from the ARD-SP and NHRC FRI programs. The Army Public Health Center collected weekly ARD-SP data from the Army’s 4 IET sites (Fort Benning, GA; Fort Jackson, SC; Fort Leonard Wood, MO; and Fort Sill, OK). ARD case criteria were oral temperature >100.5°F, recent sign or symptom of acute respiratory tract inflammation, and having a limitation in training or removal from duty. We determined ARD rates for each IET installation and for the total Army IET population using the equation (ARD cases/all trainees) × 100 trainee weeks, and used SPSS version 21 (SPSS, Inc., Chicago, IL, USA) for analyses.

**Figure 1:**
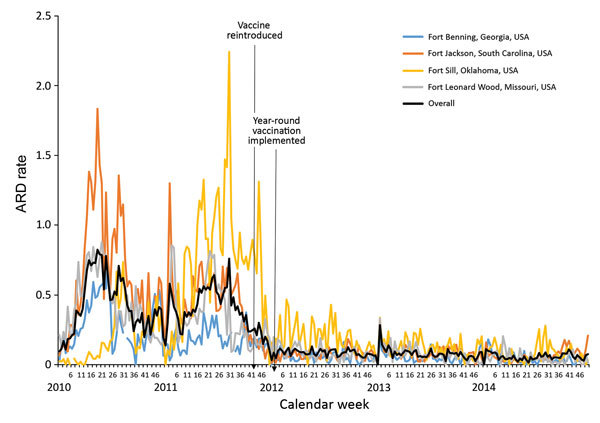
Weekly acute respiratory disease (ARD) rates by US Army initial entry training site, 2010–2014. ARD rate = (ARD cases/all trainees) × 100 trainee weeks.

An ongoing program of year-round administration of AdV-4 and -7 vaccine at Army IET sites began in November 2011. Overall, ARD rates decreased in November 2011 (9% from 2010 rates) and each subsequent year through 2014 (Table). However, in 2011, Fort Sill experienced an increase over 2010 ARD rates before rates dropped substantially in 2012 (0.75 cases/100 trainee weeks in 2011 vs. 0.19 cases/100 trainee weeks in 2012) and remained low through 2014 (Figure 1; Table). All 4 sites experienced similar declines from 2010 to 2014, ranging from 60% to 90% (Table). The combined mean ARD rate for 2010 was 7 times higher than that for 2014 (0.43 cases/100 trainee weeks vs. 0.06 cases/100 trainee weeks, respectively).

**Table:** Rate of ARD cases and percent change by year at 4 US Army initial entry training sites, 2010–2014*

Year	Average ARD rate (% change), by training site†				Overall†
	Fort Benning	Fort Jackson	Fort Sill	Fort Leonard Wood	
2010	0.29	0.67	0.20	0.43	0.43
2011	0.18 (−38)	0.44 (−34)	0.75 (+275)	0.35 (−19)	0.39 (−9)
2012	0.06 (−79)	0.08 (−88)	0.19 (−5)	0.08 (−81)	0.09 (−79)
2013	0.06 (−79)	0.09 (−87)	0.13 (−35)	0.08 (−81)	0.08 (−81)
2014	0.04 (−86)	0.07 (−90)	0.08 (−60)	0.05 (−88)	0.06 (−86)

*The adenovirus vaccine program was reintroduced in November 2011 and rate changes were studied through 2014. ARD, acute respiratory disease. †ARD rate = (ARD cases/all trainees) × 100 trainee weeks. % change calculated from last full year before vaccination (i.e., 2010).

**Figure 2:**
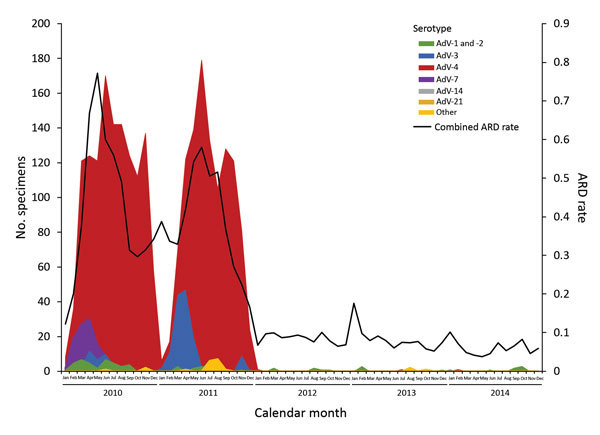
Adenovirus serotype distribution and acute respiratory disease (ARD) rate for all US Army initial entry training sites, by month, 2010–2014. ARD rate = (ARD cases/all trainees) × 100 trainee weeks.

Prior to implementation of the new vaccine in 2011, adenovirus type 4 was the predominant type at all training sites (86%), followed by types 3 (7%) and 7 (5%). After the reintroduction of adenovirus vaccine, most (71%) adenovirus-positive specimens from 2012–2014 were positive for adenovirus types 1 and 2 (Figure 2). However, appearances of adenovirus types 1 and 2 were small in scale and scattered over place and time.

## Conclusions

Reintroduction of AdV-4 and -7 vaccine had a profound effect at all Army IET sites; the combined ARD rate decreased from 0.43 cases/100 trainee weeks in 2010 to 0.06 cases/100 trainee weeks in 2014 (p<0.001), and adenovirus was identified only sporadically in ill trainees. Although a low level of ARD activity, caused by many different agents, has persisted since reestablishment of the vaccine program, the vaccine has effectively controlled the major cause of ARD at IET sites at the low cost of $150/trainee (*5*).

The increased average ARD rate for Fort Sill during 2011 was likely an anomaly associated with a lapse in the military’s long-standing routine use of benzathine penicillin G prophylaxis for group A β-hemolytic streptococcus infections coupled with a surveillance artifact introduced when Fort Sill made enhancements to their ARD surveillance program (*2*,*6*). After benzathine penicillin G prophylaxis was reintroduced, ARD rates substantially decreased in 2012, mirroring reductions observed at other IET sites after adenovirus vaccine administration.

Vaccination administration has multiple benefits. A study of US Air Force trainees with acute respiratory illness found decreased severity of systemic symptoms and reduced fever and heart rate in those who became ill after the vaccine was reintroduced (*7*). In addition, we observed an overall decrease in ARD rates and suppression of nearly all adenovirus types. Since introduction of the vaccine in 1971, many have suggested that this vaccine may have an effect on reducing ARD caused by agents other than adenovirus types 4 and 7. Recent studies have shown the AdV-4 and -7 vaccine to have a potentially broader effect, as demonstrated by decreased rates of overall febrile illness among trainees and other service members (*8*–*10*). This effect could be due to activation of innate immunity and heterotypic antibody response (*11*,*12*).

The overall observed reduction in ARD among Army IET trainees translates to substantial cost savings by reducing the probability of severe illness or death and lost training time. During the 1999–2010 lapse in adenovirus vaccine coverage, 8 adenovirus-infected service members died (*13*). Estimates showed each infection costs ≈$3,838, and each year the vaccine prevents 1 death, 1,100–2,700 hospitalizations, and 13,000 febrile infections among military recruits. Vaccination costs $150 per person, providing a net savings of ≈$20 million (*5*). Phase 3 safety studies of the vaccine established an excellent safety profile (*14*). Surveillance safety data since 2011 should be released soon and are expected to be consistent with the Phase 3 data.

The AdV-4 and -7 vaccine may have applications beyond the US military. Adenovirus outbreaks have occurred in non–US military populations and facilities where close contact and suboptimal hygiene may be present (e.g., militaries of other countries, dormitories, and healthcare facilities). In addition to the US military, populations in those and similar settings may benefit from AdV-4 and -7 vaccine (*15*).
